# Effect of evidence-based nursing practices training programme on the competency of nurses caring for mechanically ventilated patients: a randomised controlled trial

**DOI:** 10.1186/s12912-024-01869-1

**Published:** 2024-04-02

**Authors:** Sameh Elhabashy, Michiko Moriyama, Eman Ibrahim El-Desoki Mahmoud, Basem Eysa

**Affiliations:** 1 Faculty of Nursing, Cairo University, 11562 Cairo, Egypt; 2 Graduate School of Biomedical and Health Sciences, Hiroshima University, 734-8551 Kasumi, Hiroshima, Japan; 3 National Hepatology and Tropical Medicine Research Institute, Cairo, Egypt

**Keywords:** Evidence-based practice, Nursing, Training, Mechanical ventilation, Competency

## Abstract

**Background:**

Evidence-Based Practice (EBP) has been recognised worldwide as a standardised approach for enhancing the quality of healthcare and patient outcomes. Nurses play a significant role in integrating EBP, especially in Intensive Care Unit (ICU). Consequently, this study aims to examine the effect of an adapted evidence-based nursing practices training programme on the competency level of nurses caring for mechanically ventilated patients.

**Methods:**

A prospective open-label parallel 1:1 randomised controlled trial was conducted on 80 nurses caring for ICU patients at the National Hepatology and Tropical Medicine Research Institute, Egypt. The trial was carried out between November 2022 and February 2023 under the registration number NCT05721664. The enrolled nurses were randomly divided into intervention and control groups. The intervention group received the evidence-based nursing practice training programme (EBNPTP) in accordance with the Johns Hopkins EBP conceptional model, whereas the control group received traditional in-service education. Four assessments (one pre- and three post-assessments) were conducted to evaluate nurses’ competency level over time using the adapted evidence-based nursing competency assessment checklist. The primary endpoint was an increase the competency levels among nurses caring for mechanically ventilated patients.

**Results:**

The current study results revealed statistically significant differences between intervention and control groups in relation to their level of competency across the three post-assessments, with (*p* <.001). The study also demonstrated that the nurses’ competency level continued to decline significantly over time, with (*p* <.001). Additionally, a significant correlation was found between the nurses’ pre-assessment and educational level, acting as independent variables (predictors), and the third endpoint assessment (*p* <.01), indicated by multiple linear regression.

**Conclusion:**

The EBP training programme demonstrated a significant increase in the nurses’ level of competency compared with traditional in-service education. This suggests that by training the nurses in various settings with the essential skills and knowledge for EBP, their competency level can be enhanced, leading to the delivery of effective care and improving patient outcomes. However, the long-term sustainability of the EBP adoptions was insufficient; further studies are needed to investigate the factors that affect the durability of EBP adoption.

**Trial registration:**

The study was registered with Clinical Trials.gov (Registration # NCT05721664) on 10/02/2023.

**Supplementary Information:**

The online version contains supplementary material available at 10.1186/s12912-024-01869-1.

## Background

Evidence-based practice (EBP) is a universal fundamental approach for delivering standardised care based on the most recent scientific evidence to enhance healthcare quality [1]. EBP is a problem-solving method for making effective, safe clinical decisions as a foundation for improving patient outcomes, as it bridges the theory-to-practice gap and delivers innovative patient care, while also reducing healthcare costs and encouraging lifelong learning [2]. Nurses play a crucial role in maximising the efficiency of healthcare services. Furthermore, they directly interact with patients, particularly in Intensive Care Units (ICUs) [3]. Therefore, healthcare organisations should always strive to make it easier for frontline nurses to use the best evidence in their everyday practices and overcome obstacles that may impede the implementation of the evidence [4]. EBP in healthcare is not a novel concept; Florence Nightingale introduced EBP to nursing in 1858 [5]. The concept of EBP changed as the nursing profession evolved and expanded significantly over the past few decades [6].

Care for critically ill patients necessitates a high level of competency [7]. In the ICU, mechanical ventilation (MV) is the most frequently utilised treatment modality [8]. Although MV aids in the survival of patients with respiratory compromise, it frequently results in a number of complications if they do not receive adequate nursing care [9]. Since the primary purpose of EBP is to address healthcare issues that contribute to higher mortality and morbidity rates, we have chosen to focus on ventilator-associated pneumonia (VAP) as it is a predominant complication among MV patients in Egypt. The incidence of VAP in Egypt ranges from 16 to 75% [10, 11], which is a significantly higher incidence compared to other regions, as the incidence of VAP globally is 15.6%, with rates of 13.5% in the United States, 13.8% in Latin America, and 16.0% in the Asia-Pacific region [12, 13]. Additionally, the survival rate among VAP patients in Egypt ranges from 58.3 to 31.8% [11, 14], whereas the global survival rate of VAP typically falls between 50% and 75% [15], which is also considered greater than the rate observed in Egypt. This high incidence of VAP and low survival rates in Egypt may indicate a lack of EBP in nursing practice. Due to inadequate nursing practices, particularly in the care of patients with MV, several studies recommend extensive training for nurses [16–18].

Therefore, we hypothesised that nurses who received an evidence-based nursing practice training programme (EBNPTP) (μ1) demonstrate a sustainable higher increase in their level of competency than those who received the usual traditional in-service education (μ2) in caring for mechanically ventilated patients. (H1: μ1 > μ2). This study aims to examine the effect of a designed EBNPTP on the competency level of nurses caring for patients on MV in selected ICUs in Egypt.

## Conceptual framework

**Fig. 1:**
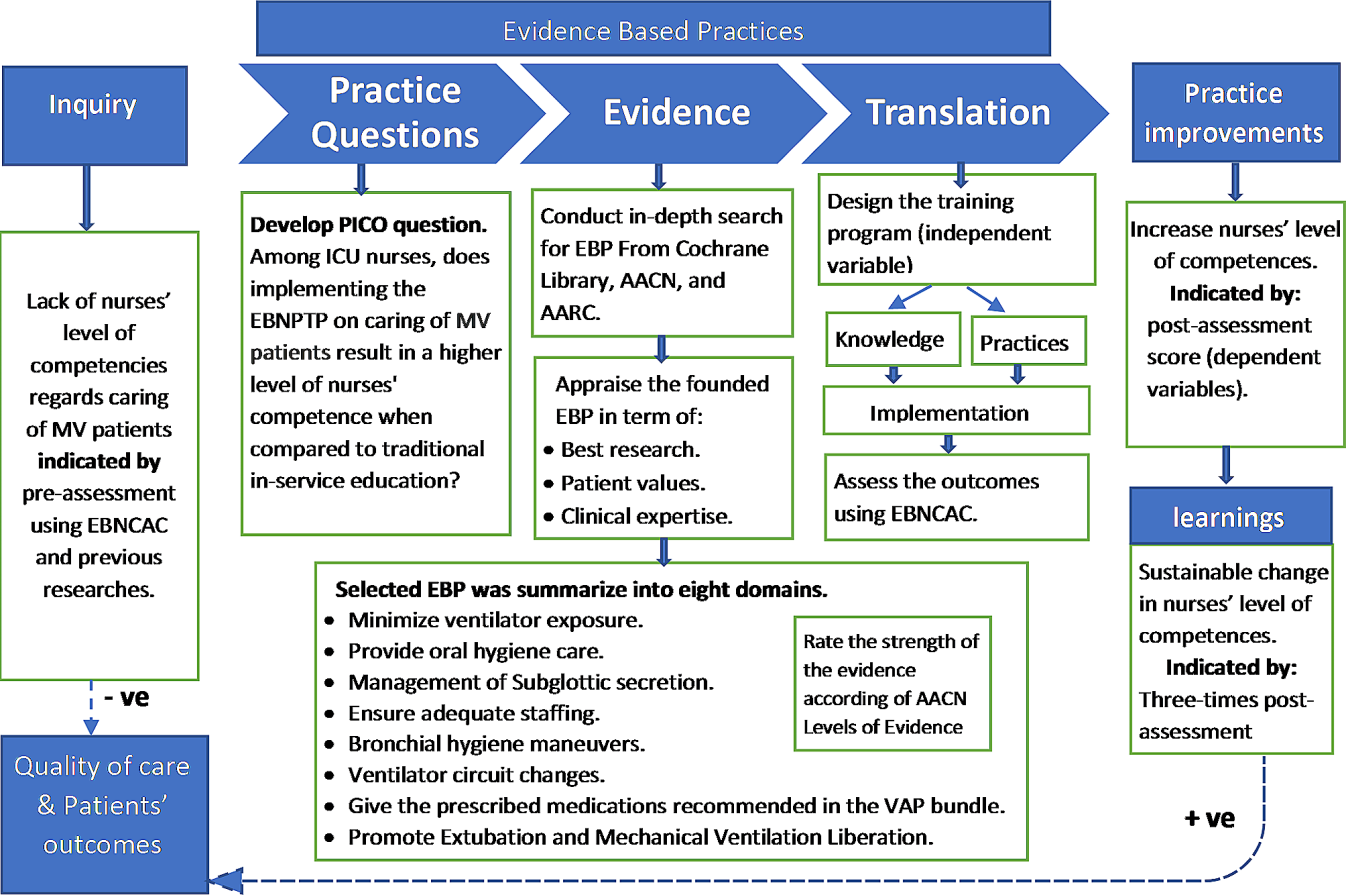
Conceptual Framework of this study EBNCAC: Evidence-Based Nursing Competency Assessment Checklist; AARC: American Association for Respiratory Care; AACN American Association of Critical-Care Nurses; EBNPTP: Evidence-Based Nursing Practice Training Programme; MV: Mechanical Ventilator; ICU: Intensive Care Unit; EBP: Evidence-Based Practices; VAP: Ventilator-Associated Pneumonia

The revised Johns Hopkins Evidence-Based Practice (JHEBP) Model [4] was selected as a systematic and efficient approach to implementing an evidence-based programme into practice in this study Fig. 1. The JHEBP model encompasses four essential components: inquiry, practice, practice improvements, and learning. Nurse performance is considered the most typical determinant and predictor of the quality of care and patient outcomes [19]. Due to a lack of nurses’ level of competency regarding caring for MV patients, the quality of provided care and patients’ outcomes are negatively impacted [16–18]. As an independent variable, we designed the EBP training programme for ICU nurses based on the JHEBP method. The EBP training programme contains eight domains listed in Fig. 1, which meet the educational needs of nurses in terms of both knowledge and practices. The ultimate objective of the training is to provide a positive, sustainable change in nurses’ level of competencies, thereby improving patient outcomes [20]. The JHEBP Model defines learning as a sustainable change in candidates’ behaviour. Therefore, the post-assessment of nurses’ competency as a dependent variable was measured three times at one-month intervals to evaluate the over-time change compared to the baseline pre-assessment and control group.

## Methods

### Trial design

The current study was a prospective open-label parallel 1:1 randomised controlled trial. This study’s protocol was developed following the Standard Protocol Items Recommendations for Interventional Trials [21]. The study was conducted between November 2022 and February 2023 at the National Hepatology and Tropical Medicine Research Institute (NHTMRI) in Cairo, Egypt, in accordance with the Consolidated Standards of Reporting Trials (CONSORT) guidelines [22].

### Participants sampling and study setting

The study was conducted in adult ICUs at the NHTMRI, Cairo, Egypt. The total capacity of the ICU is 14 beds, divided into two sections. One section was allocated for nurses who were assigned as an intervention group, while the other section was allocated for nurses who served as a control group. By randomly assigning nurses to each group and ensuring they work in separate ICU sections, the risk of contamination bias is reduced. These sections are comparable in terms of patient flow, equipment availability, and the number of working nurses. The total number of nurses in the selected setting is 94. All selected nurses met specific inclusion criteria: Willing to participate in this research, hold the existing position for at least three months; this criterion ensures that participants have had sufficient time to become familiar with their roles and responsibilities, allowing for a more accurate assessment of any changes or improvements in competency resulting from the EBP training programme. Additionally, participants were required to have at least two years of critical care experience, ensuring they possess a solid foundation of knowledge and skills necessary for caring for MV patients. This experience enhances the credibility of their feedback on the training programme’s effectiveness. Nurses intending to leave their jobs within the study period (four months) were excluded.

### Sample size calculation

The sample size of 80 nurses was estimated by G power software V.3.1.9.4 (Psychonomic Society, Madison, Wisconsin, USA) with α = 0.05, power (1-β err prob) = 0.80, effect size = 0.56, and confidence level of 0.95. In terms of statistical power and effect size, the sample size chosen for our study was deemed adequate based on the previous study that studied the impact of an education programme on the performance of nurses providing care for patients on MV [23].

### Randomisation and allocation

After verifying the eligibility criteria, the enrolled nurses were randomly divided into intervention and control groups. A simple random sample was generated by a lottery method. The eligible nurses were assigned a number, and each number was written and placed in a small opaque envelope. Then random selection and allocation were performed sequentially for the intervention and control groups. Randomisation and allocation were conducted by an unaffiliated third party.

### Outcomes

#### The primary endpoint

The primary outcome was an ‘increase in the competency level’ of nurses caring for MV patients, measured by the Evidence-Based Nursing Competency Assessment Checklist (EBNCAC) over three months after receiving EBNPT, aiming to comprehensively evaluate the effectiveness and durability of the provided EBNPTP and to ensure the stability of results.

### Measurement tools

EBNCAC is a structured observational checklist assessing nurses’ competency developed by the researcher and compiled from evidence-based clinical guidelines listed in Fig. 1. Encompassing 74 items, the checklist covers eight domains addressed in the Evidence-Based Nursing Practice Training Programme (EBNPTP). The tool was structured based on various sources, including the American Association of Critical-Care Nurses (AACN), the American Association for Respiratory Care (AARC), the National Institutes of Health (NIH), and the Cochrane Library [24–27]. The responses of nurses to each item were graded on a scale of 2 to 0. “2 = performed correctly and satisfactory”, “1 = performed but unsatisfactory,” and “0 = not performed”. The total score ranged from 0 (lowest) to 148 (highest). Assessors were the charge nurses in the selected ICUs; they directly observed the nurses’ performance while participants cared for the MV patients. Based on the total score of 148, the scoring level was divided into three categories: High (> 120 / >80%), Moderate (74–120 / 50–80%), and Low (74 / 50%).

### Validity and reliability

Content and scope validity for EBNCAC were determined utilising the Lawshe method [28]. The tool was revised by five experts in critical care medicine and nursing. Following the Subject Matter Expert (SME) ratings, the content validity ratio (CVR) was calculated for each item using the formula (ne–N/2)/(N/2), where ne represents the number of SMEs indicating “essential” and N denotes the total number of SMEs. The Content Validity Index (CVI) was then calculated by averaging the CVRs across all items, resulting in a value of 0.98 (72.57/74). The scale’s reliability was assessed using internal consistency (Cronbach’s alpha) for all items in the EBNCAC. The calculated Cronbach’s alpha for the EBNCAC was 0.721.

### Evidence-based nursing practices training programme (EBNPTP) for the intervention group

BNPTP pertains to the care of MV patients. This is an integrated theoretical and clinical course for one week (30 h) designed by the researchers. To ensure the validity and reliability of the provided EBNPTP, it was formulated based on the latest research findings in the relevant areas of this study, such as those from the AACN and Cochrane Library [24, 27]. Furthermore, it underwent review by three professional experts in critical care nursing and medicine to ascertain content validity. Also, three ICU nurses were enlisted to conduct a pre-test assessing the feasibility and acceptability of the training programme and the tool. Necessary revisions were made based on their feedback, and these nurses were excluded from the sample frame for enrollment. We standardised the delivery of the EBNPTP to enhance reliability by providing clear instructions to facilitators and conducting training sessions in a controlled and consistent manner. Finally, setting amenities and nurses’ and patients’ preferences were considered as it is a necessity of EBP. During the training week, the nurses in the intervention group (*n* = 40) were divided into two equal groups. They were scheduled to exchange their working days in the ICU with their training times to prevent any interruption of workflow in the ICU. The nurses’ considerable clinical experience enabled them to effectively fulfil the objectives of the condensed course.

### Control group

The control group received traditional in-service education on a regular basis from the quality management department and nursing office. Usually, the educational content was provided in accordance with the educational needs of nurses. Routine clinical guidance was usually provided in real clinical settings. Additionally, periodic supplementary sessions were organised to address significant incidental clinical issues encountered by nurses.

### Data collection procedure

After obtaining the informed consent, recruitment started in November 2022, and baseline pre-assessment was conducted in November 2022 using the EBNCAC. It serves as the initial assessment before the EBNPTP intervention, which was provided in one week at the end of November 2022. The first post-assessment was conducted immediately after the EBNPTP at the beginning of December 2022. It measures the immediate impact of the EBNPTP using the EBNCAC. The second post-assessment took place one month after the first assessment in January 2023. The Third Post-assessment occurred in the second half of February 2023, one month after the second post-assessment and three months after the EBNPTP, serving as the endpoint assessment. Considering that each assessment was held within one week, the interval between the four assessments was one month.

### Data analysis

This study utilized a per-protocol analysis. Statistical Package for Social Sciences (SPSS) V.23.0 (IBM, New York) was used for analysis. Data were expressed using mean and standard deviation (SD). The normal data distribution was examined using Shapiro-Wilk’s test, histograms, box plots, and normal Q-Q plots for both the control and intervention groups with (*p* >.05). The two groups were compared by a two-way repeated measure of ANOVA. Multiple linear regression was applied as a regression model to test the effect of the study predictors on the endpoint third post-assessment. Finally, the effects of demographic characteristics on the baseline pre-assessment and endpoint of the third post-assessment were determined utilising a t-test and one-way ANOVA. The significance level was set at (*p* <.05).

## Result

**Fig. 2:**
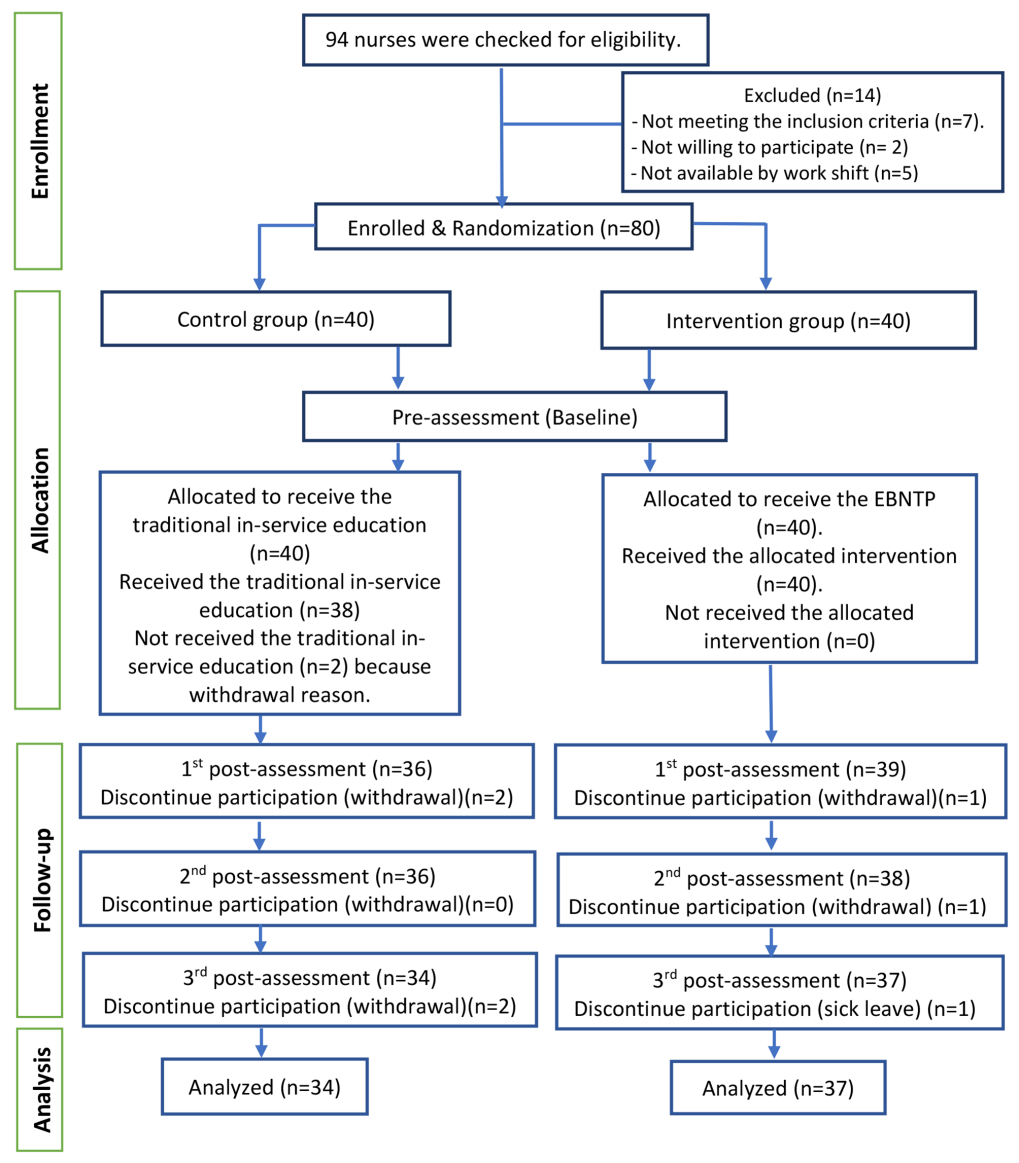
CONSORT flow diagram shows the participation in this study

Out of 94 nurses, 14 were excluded as they did not meet the eligibility criteria. Eighty nurses were equally allocated into the intervention group and control group. Ultimately, the third post-assessment data was carried out for 71 nurses (intervention, *n* = 37; control, *n* = 34). The reasons for dropout throughout the follow-up using three post-assessments are reported in Fig. 2. The scores for nurses’ competency subscales across the control and intervention groups at the four observation times are depicted in supplementary material 1.

**Table 1:** Demographic characteristics of the enrolled participants at baseline (*n* = 80)

Demographic characteristics	Groups		Total (*n* = 80) *n* (%)	*p*
	Control (*n* = 40) *n* (%)	Interventional (*n* = 40) *n* (%)		
**Gender**:				0.785 ^a^
Male	8 (20.0)	9 (22.5)	17 (21.3)	
Female	32 (80.0)	31 (77.5)	63 (78.8)	
**Years of experience in ICU**:				0.463 ^a^
1–10	23 (57.5)	21 (52.5)	44 (55)	
> 10–20	8 (20.0)	13 (32.5)	21 (26.3)	
> 20–30	8 (20.0)	6 (15.0)	14 (17.5)	
> 30–40	1 (2.5)	0 (0)	1 (1.3)	
Mean ± SD	10.4 ± 8.33	10.2 ± 7.80	10.3 ± 8.02	0.945 ^b^
**Educational level**:				0.192 ^a^
Diploma nurses	21 (52.5)	25 (62.5)	46 (57.5)	
Technical nurses	15 (37.5)	8 (20.0)	23 (28.8)	
Bachelor’s nurses	4 (10.0)	7 (17.5)	11 (13.8)	
**Age**:				0.855 ^a^
20–30	18 (45.0)	18 (45.0)	36 (45.0)	
> 30–40	11(27.5)	12 (30)	23 (28.8)	
> 40–50	7 (17.5)	8 (20.0)	15 (18.8)	
> 50–60	4 (10.0)	2 (5.0)	6 (7.5)	
Mean ± SD	33.4 ± 9.79	33 ± 9.15	33.2 ± 9.42	0.842 ^b^

^a^ Chi-square. ^b^ t-test

At baseline, there were no significant differences between the groups in regard to their demographic characteristics Table 1. Most participants were female (78.8%), and more than half were diploma nurses (57.5%). Their mean age and length of experience at the ICU were 33.2 years old and 10.3 years, respectively.

**Fig. 3:**
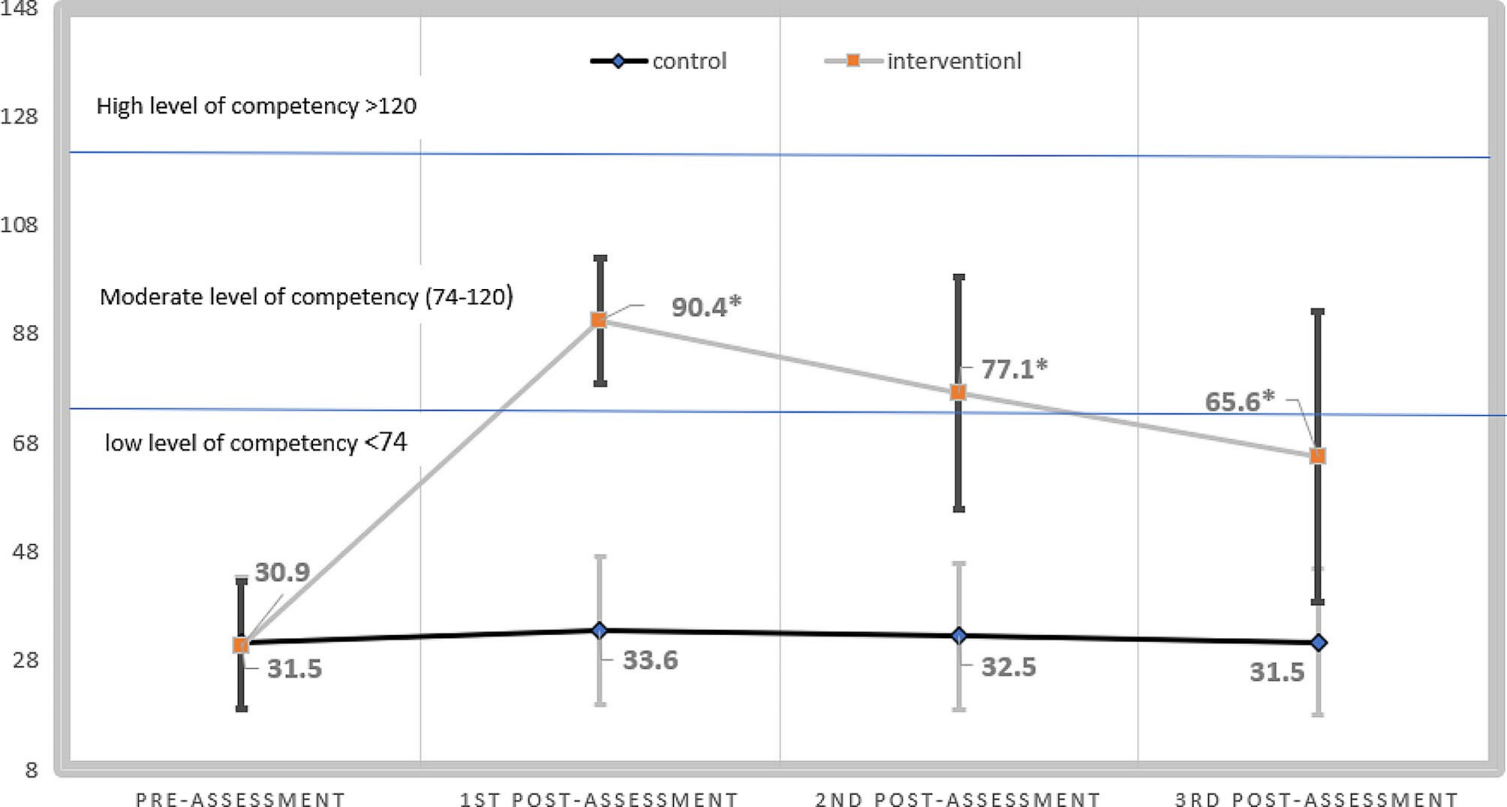
Comparison between nurses’ level of competency over the four times of measurements *P <.001

The stacked line chart in Fig. 3 depicts the chronological change in nurses’ competency level measured by EBNCAC among the two groups, revealing a low level of competency at the baseline with no significant difference between the two groups (*p* =.81). The highest level of competency was at the first post-assessment score among the interventional group, with a mean score of (90.4 ± 11.55). The mean score declined steadily until the third post-assessment reaching a mean score of (65.6 ± 26.70). The control group demonstrated a low level of competency along with the four-time assessments, with mean scores ranging from 31.5 to 33.67 out of 148. Statistically significant differences were observed between the groups among the three post-assessments, with (*p* <.001).

**Table 2:** Nurses’ competency scores over four times of assessments between control and interventional groups

Measurements	Groups			Two-way repeated measures ANOVA						
	Control (*n* = 34) Mean ± SD	Interventional (*n* = 37) Mean ± SD			Sum of squares	df	Mean Squares	F	*p*	η2
Pre-assessment	31.55 ± 12.01	30.89 ± 11.56		Group (Between)	80404.23	1	80404.236	159.88	<.001	0.699
1st post-assessment	33.67 ± 13.51	90.40 ± 11.55		Time (within)	36882.69	1.660	22217.231	64.247	<.001	0.482
2nd post-assessment	32.58 ± 13.29	77.13 ± 21.31		Time × Group	32411.17	1.660	19523.696	56.458	<.001	0.450
3rd post-assessment	31.52 ± 13.51	65.64 ± 26.70								

SD: standard deviation; η2: partial eta squared

According to Table 2, there is a statistically significant difference within groups in terms of pre, first, second, and third assessments in relation to the nurses’ competency level (*p* <.001). In addition, there is a statistically significant difference between groups regarding the nurses’ competency level (*p* <.001), specifically in the three post-assessment phases. with a considerable high estimated effect size (η2 = 0.699). Finally, there is an interaction effect between the measurements in time and group (*p* <.001). Therefore, the result of the Two-way repeated measures ANOVA supported our hypothesis that the EBNPTP significantly increased nurses’ level of competency.

**Table 3:** The predictors on 3rd post-assessment (Endpoint observation) by multi-linear regression

Model			B	β	t	Sig.	R	R Square	Adjusted R Square	F	*p*
Dependent	Independent variables										
3rd post-assessment (Endpoint)	Age	0.003	0.001	0.010	0.992	0.834	0.696	0.667	24.36	< 0.001	
	gender	5.592	0.084	1.193	0.237						
	years of experience in ICU	− 0.443	− 0.133	-1.083	0.283						
	Educational level	14.124	0.377	4.907	< 0.001						
	Pre-assessment (Baseline)	0.530	0.227	3.236	0.002						
	Control-Interventional groups	35.696	0.657	9.489	< 0.001						

β, Standardized regression coefficient; B, Unstandardized regression coefficient; ICU, Intensive care unit

1st and 2nd post-assessment were excluded from the regression model because a VIF was above five to avoid render other significant variables redundant

Table 3 presents the results of multiple linear regression models, which reveal the effect of these independent variables on the endpoint observation. The study revealed that pre-assessment (B = 0.53, *p* =.002), educational level (B = 14.12, *p* <.001), and control-intervention groups strongly predicted improvement of the third post-assessment (Endpoint) (B = 35.69, *p* <.001). The adjusted R square was (0.667), indicating that the model could account for approximately 66.7% of third post assessment improvement.

**Table 4:** Influence of demographic characteristics on the pre-assessment and 3rd post-assessment

Demographic characteristics	*N*	Pre-assessment as a baseline data				3rd post-assessment as an endpoint				
		Mean ± SD	t/F	*p*-value		Mean ± SD			t/F	*p*-value
Gender										
male	15	31.53 ± 14.02	.11^a^	0.90		42.00 ± 17.80			-1.53^a^	0.13
female	56	31.12 ± 11.14				51.26 ± 29.19				
Age			1.18^b^	0.32					2.03^b^	0.11
20–30	31	32.77 ± 10.25				56.96 ± 29.85				
> 30–40	20	30.80 ± 12.09				47.85 ± 25.64				
> 40–50	14	26.57 ± 14.65				41.64 ± 25.08				
> 50–60	6	35.33 ± 8.71				32.50 ± 10.42				
Years of experience in ICU										
1–10	38	32.57 ± 12.62	.44^b^	0.72		55.13 ± 29.77			2.72^b^	0.051
> 10–20	19	30.31 ± 9.92				50.47 ± 25.87				
> 20–30	13	29.00 ± 11.98				32.84 ± 12.23				
> 30–40	1	25.00 ± 0				20.00 ± 0				
Level of education										
Diploma Nurses	40	30.15 ± 10.32	1.12^b^	0.33		41.15 ± 18.13	]*	]**	9.41^b^	< 0.001
Technical institute	21	30.80 ± 14.38				50.85 ± 32.46				
Bachelor’s degree	10	36.30 ± 10.42				78.70 ± 28.03				

^a^*t*- t e s t s, ^b^*F*- t e s t s

Bonferroni *p <.05, ** *p* <.001

SD: Standard deviation; ICU: Intensive care unit

Table 4 indicates significant differences between nurses’ level of education and the scores of the third post-assessment (F = 9.41, *p* <.01). The 3rd post assessment score was significantly higher for bachelor’s degree nurses than for diploma (*p* <.001) and technical institute nurses (*p* <.05), while no significant differences between nurses’ pre / third post-assessments related scores in term of their gender, age, and years of experience.

## Discussion

To the of our knowledge, this is the first randomised controlled trial (RCT) in Egypt and the Middle East that aimed to quantitatively examine the effect of the EBNPTP caring for MV patients and assess the sustainability effect over time. The current study showed that the nurses who received the EBNPTP demonstrated a higher level of competency than those who did not. Congruently, several studies revealed that nurses’ level of competency was significantly improved by attending an EBP educational programme [29–32]. These findings strongly advocate for the widespread adoption of EBP utilisation to enhance nurses’ competency levels. Conversely, previous research has suggested that although EBP enhances nurses’ practices, it does not have a significant impact on their knowledge and attitude [33].

The improvement in nurses’ level of competency after receiving the EBNPTP can be attributed to a number of factors, including an increase in their job satisfaction, a sense of confidence, and an increase in their knowledge and skills, which provides them with a rationale for each specific task they perform. These provided justifications align with previous research [4, 31]. From another point of view, this finding underscores deficiencies in baseline nurses’ understanding of EBP and its inadequate integration into their clinical practices. Moreover, the findings hint at the ineffectiveness of traditional in-service education delivered to nurses. Indicates the importance of substituting traditional in-service education with EBP training programmes and wide use of such programmes across different nursing domains.

In terms of the sustainability effect of EBNPTP among the intervention group, this study demonstrated that the mean scores of the nurses who received EBNPTP decreased significantly over time, indicating a lack of sustainability in the nurses’ level of competency. Even though the third post-assessment score for the intervention group was the lowest, it was still significantly two times higher than the baseline per-assessment score for the same group and the average scores of the control group. Similarly, Chu et al. (2019) [34] found that the experimental group’s scores substantially improved more than the control group one month after the training. However, both groups’ results declined; still, the experimental group performed better than the control group, indicating the effectiveness of the EBNPTP. Short-term initiatives of EBP education are likely to be successful. Nevertheless, there is little evidence regarding these initiatives’ sustainability [34, 35]. Conversely, other studies confirmed that the participants’ EBP competencies were significantly improved and maintained over time [36, 37].

The rationale for the lack of sustainability of nurses’ competencies can be attributed to nurses’ attitudes, resistance to change, lack of motivation, inadequate commitment, insufficient clinical supervision, stressful work environment, and workload. This finding is consistent with previous studies [31, 36]. The lack of sustainability may also result from insufficient availability of essential equipment and supplies required for conducting EBP. Hence, it is strongly advised to provide effective clinical supervision for nurses in parallel with implementing EBP and to encourage nurses to adopt EBP consistently. Also, ensuring the constant availability of the necessary equipment.

Regarding the variables that affect the competency level of the nurses, multi-linear regression analysis revealed that a higher educational level was associated with a higher level of nurses’ competency. This result could be attributed to the higher level of skills and knowledge that baccalaureate-educated nurses possess; they are also more confident and take the initiative to update their knowledge, which makes them more capable of performing competently. On the same line, Hashish et al. (2020) [38] illustrated that experienced and baccalaureate nurses are more likely to access more resources, power, and knowledge that enable them to undertake autonomous and EBP than diploma programmes. Notably, the majority (approximately 90%) of nurses in Egypt hold diplomas, while only 6–8% hold bachelor’s degrees [39].

Furthermore, the study found that the baseline pre-assessment was an independent predictor and showed a significant relationship with the third assessment. This may be due to the fact that those who demonstrated in a particular way will continue to do so, and the nurses’ performance depends on their previous accumulated knowledge. This finding aligns with previous research which stated that nurses with a higher baseline competency level are likely to be more confident in implementing EBP [40]. However, the study revealed that nurses’ gender, age, and years of experience did not significantly affect their competency level before and after EBNPTP. This finding may be due to the reliability and accessibility of the provided EBNPTP, which was available to all nurses irrespective of these demographic variations, thus suggesting that EBNPTP implementation in the future could be beneficial for all nurses, regardless of these demographic characteristics. Consistently, Stokke et al. (2014) stated that none of the nurses’ demographic characteristics were found to be correlated with the implementation of EBP [41]. Since nurses’ attitude and motivation reflect their professional values and performance, prior research emphasises the importance of enhancing these factors when encouraging nurses to adopt EBP into their practices [42].

## Conclusion

The current RCT is the first in Egypt and the Middle East to investigate the effect of an EBP training programme on the competency of nurses caring for MV patients and to assess the effect’s sustainability over time. In accordance with the research hypothesis, the EBP training programme demonstrated a significant increase in the nurses’ level of competency compared with traditional in-service education. This highlights the potential for widespread adoption of EBP across various areas of nursing to enhance the quality of care provided. However, the efficacy of the EBP training programme was found to be unsustainable over time. Addressing this challenge requires integrating the EBP training programme as material for job development and remuneration training for nurses to enhance its long-term effectiveness, ongoing monitoring of nurses’ performance, and further assessment of the contributing factors. The baseline competency and educational level of nurses correlate significantly with their performance. Consequently, the difficulties in dealing with nurses with varying levels of education and diminishing competencies persisted. This suggests the need for customised training programmes based on nurses’ baseline competency levels and educational backgrounds, as well as facilitating peer support and mentorship.

### Limitations of the study

The enrolled participants were selected from a limited number of nurses. In addition, when the researchers estimated the sample size, the dropout rate was not considered, which may affect the power of the sample size; the dropout rate was 11%. The study included a small number of allocated bachelor nurses. Another limitation is that only three months were the duration to assess the sustainability effect of EBNPTP over time, which may be a short period. Additionally, data was collected from only one hospital.

### Recommendations

According to the current study’s findings, we strongly suggest implementing EBP in nursing practices through elevating awareness and delivering extensive training for nurses across different settings. By equipping nurses with the necessary skills and knowledge for EBP, their competency can be enhanced, thus contributing to improved patient outcomes. Additionally, assign highly educated nurses to critical care settings requiring advanced care. Also, to sustain the implementation of EBP, we recommend providing effective clinical supervision. Furthermore, we propose a larger-scale evaluation of the impact of EBP implications in a variety of nursing specialisations. We therefore strongly advise evaluating the effects of EBP on patient outcomes. In addition, it is important to assess the factors or obstacles that may affect the application of EBP in nursing and to maintain its sustainability, as well as to identify the gap between education and practice using qualitative and quantitative research methods.

### Electronic supplementary material

Below is the link to the electronic supplementary material.

Supplementary Material 1

## Data Availability

The generated tool of data collection EBNCAC, intervention training programme EBNPTP and raw data of this study are available from the corresponding author upon request.
