# Supplementary material for: Effect of evidence-based nursing practices training programme on the competency of nurses caring for mechanically ventilated patients: a randomised controlled trial
Source: BMC Nurs. 2024 Apr 2;23:225. doi: 10.1186/s12912-024-01869-1 (PMC10986015; doi:10.1186/s12912-024-01869-1)
Supplement: Supplementary file 1 — Supplementary Material 1 [file 12912_2024_1869_MOESM1_ESM.docx]

**Supplementary material 1**

The scores for nurses' competency subscales across the control and intervention groups at the four observation times (n=71).


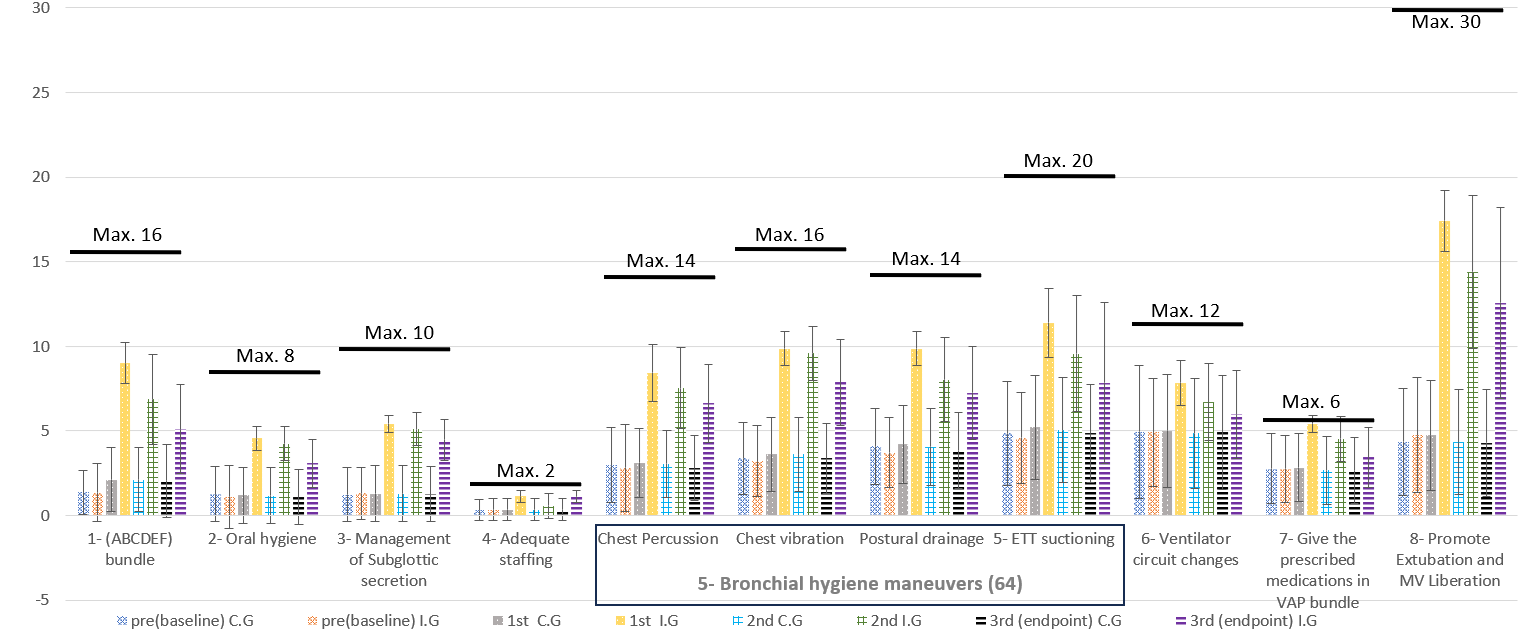


Max.= maximum score

Eight domains with eleven competency subscales comprised a total of 74 items, with a maximum achievable score of 148. Concerning the initial pre-assessment observations, the lowest scores in the subscale focused on implementing the ABCDEF bundle were attained by nurses in both the control and interventional groups, scoring (1.38 + 1.3, 1.35 + 1.7), respectively, out of a possible 16. Conversely, the highest subscale scores were associated with administering prescribed medications, whereby nurses in the control and interventional groups achieved (2.76 + 2, 4.75 + 3.4), respectively, out of 6. Regarding the third post-assessment (endpoint), as depicted in figure 4, the lowest score within the interventional group still pertained to the utilisation of the ABCDEF bundle subscale, with a mean score of 5.10 + 2.6 out of a maximum of 16. Conversely, the highest score was also linked to administering prescribed medications, with a mean score of 3.45 + 1.7 out of 6.
